# Downhill running affects the late but not the early phase of the rate of force development

**DOI:** 10.1007/s00421-022-04990-8

**Published:** 2022-07-06

**Authors:** Giorgio Varesco, Giuseppe Coratella, Vianney Rozand, Benjamin Cuinet, Giovanni Lombardi, Laurent Mourot, Gianluca Vernillo

**Affiliations:** 1 Inter-University Laboratory of Human Movement Biology (EA 7424), UJM-Saint-Etienne, Université de Lyon, 42023 Saint-Etienne, France; 2 Department of Biomedical Sciences for Health, Università degli Studi di Milano, Building 2, via G. Colombo 71, 20133 Milan, Italy; 3 Laboratory of Experimental Biochemistry and Molecular Biology, IRCCS Orthopedic Institute Galeazzi, 20161 Milan, Italy; 4 Department of Athletics, Strength and Conditioning, Poznań University of Physical Education, 61-871 Poznań, Poland; 5 Prognostic Factors and Regulatory Factors of Cardiac and Vascular Pathologies (EA3920), Exercise Performance Health Innovation (EPHI) Platform, University of Bourgogne Franche-Comté, 25000 Besançon, France; 6 Division for Physical Education, Tomsk Polytechnic University, Tomsk Oblast, 634050 Russia

**Keywords:** Eccentric exercise, Fatigue, Quadriceps muscle, Running, Trail running

## Abstract

**Purpose:**

This study aimed to evaluate the acute changes in the knee extensors maximum voluntary isometric contraction force (MVIC), rate of force development (RFD), and rate of EMG rise (RER) following a bout of downhill running.

**Methods:**

MVIC and RFD at 0–50, 50–100, 100–200, and 0–200 ms were determined in thirteen men (22 ± 2 yr) before and after 30 min of downhill running (speed: 10 km h^−1^; slope: − 20%). *Vastus lateralis* maximum EMG (EMG_max_) and RER at 0–30, 0–50, and 0–75 ms were also recorded.

**Results:**

MVIC, RFD_0–200_, and EMG_max_ decreased by ~ 25% [Cohen’s *d* = − 1.09 (95% confidence interval: − 1.88/− 0.24)], ~ 15% [*d* = − 0.50 (− 1.26/0.30)], and ~ 22% [*d* = − 0.37 (− 1.13/0.42)] (all *P* < 0.05), respectively. RFD_100–200_ was also reduced [− 25%; *d* = − 0.70 (− 1.47/0.11); *P* < 0.001]. No change was observed at 0–50 ms and 50–100 ms (*P* ≥ 0.05). RER values were similar at each time interval (all *P* > 0.05).

**Conclusion:**

Downhill running impairs the muscle capacity to produce maximum force and the overall ability to rapidly develop force. No change was observed for the early phase of the RFD and the absolute RER, suggesting no alterations in the neural mechanisms underlying RFD. RFD_100–200_ reduction suggests that impairments in the rapid force-generating capacity are located within the skeletal muscle, likely due to a reduction in muscle–tendon stiffness and/or impairments in the muscle contractile apparatus. These findings may help explain evidence of neuromuscular alterations in trail runners and following prolonged duration races wherein cumulative eccentric loading is high.

## Introduction

Trail running is an endurance-oriented discipline consisting of prolonged running on rough terrain with an alternation of uphill and downhill segments (Scheer et al. 2020). The popularity of these events has seen an exponential increase worldwide (Hoffman et al. 2010), and the combination of long distance and large sections of downhill running has been hypothesized to induce deleterious alterations to the performance of the neuromuscular system (Millet et al. 2011; Saugy et al. 2013; Temesi et al. 2014; Vernillo et al. 2019). Particularly during downhill running, the considerable role of the knee extensor muscles in energy absorption and dissipation (Khassetarash et al. 2020) results in high force eccentric muscle actions (Vernillo et al. 2017, 2020a), inducing muscle fatigability (Giandolini et al. 2016b; Bontemps et al. 2020). Accordingly, the research investigating the trail running performance identifies the downhill segments as particularly deleterious for the muscle function (Martin et al. 2004; Giandolini et al. 2016a; Ehrström et al. 2018).

Endurance running, both on roads and on trails, involves the repetitive activation of large muscle masses of the lower limbs for prolonged periods of time (Novacheck 1998). In addition, each step requires the ability to rapidly develop force (Nicol et al. 2006), a key aspect for trail running where technical segments and elevation changes could require a rapid muscle force generation. Much of the research investigating the influence of downhill running-induced fatigability has focused on the decline in maximum voluntary isometric contraction force (MVIC) (Giandolini et al. 2016b; Bontemps et al. 2020). Nonetheless, the capacity to develop a high amount of force in a very short amount of time is considered functionally more relevant than MVIC (Maffiuletti et al. 2016) and was recently proposed as an indicator of neuromuscular fatigue (D’Emanuele et al. 2021). Such a capacity is defined as the rate of force development (RFD) and can be assessed by measuring the slope of the force–time curve during short (~ 1 s) isometric impulsive contractions over a window of 200 ms, as suggested by Maffiuletti et al. (2016). Two phases, the early and late phase, have been identified to better understand all the neural and structural factors that could contribute to the RFD (Maffiuletti et al. 2016). Specifically, the early phase (the firsts 50 ms from the onset of the contraction) has been associated with the initial motor unit recruitment and firing rates (Klass et al. 2008), whereas maximum strength and other structural factors, such as muscle contractile properties (Aagaard et al. 2002) and muscle architecture (Coratella et al. 2020), influence the late phase (last 100 ms of the 200 ms time-window).

Accordingly, the analysis of RFD may be used to detect the fatigue-induced changes after an exercise, and an examination of the early and late phases may reflect the neural and structural fatigue-induced changes, respectively (Aagaard et al. 2002; Boccia et al. 2017). Furthermore, the RFD analysis can be complimented by the analysis of the rate of surface electromyography (EMG) signal rise (RER) to further detail the contribution of neural factors to RFD as a non-invasive surrogate of the capacity to deliver high-frequency motor unit bursts (i.e., lower maximal discharge frequency and incidence of doublet discharge during fast contractions) (Klass et al. 2008; Del Vecchio et al. 2019). Thus far, it has been shown that the RFD is impaired after level running events [e.g., half-marathon (Boccia et al. 2018)], whereas a limited number of studies have investigated the ability of rapidly develop force after downhill running. Specifically, Lima et al. (2020) examined the changes in RFD in the knee extensors immediately after a downhill run, reporting a ~ 20% decrease in peak RFD following a 30-min downhill running at 10 km h^−1^ and − 15% incline. However, neither the different phases of the force–time curve of RFD nor the RER were investigated, limiting the understanding of possible factors negatively affecting RFD following downhill running.

Therefore, the present study aimed to investigate the changes in the knee extensors RFD after a downhill run. If an effect of downhill running was observed on maximal force and RFD (200 ms window), the various phases of RFD, accompanied by the EMG rise, were investigated to determine the etiology (neural vs. muscular) of the fatigue-induced changes on the functional capacity of the neuromuscular system to develop a high amount of force in a very short period of time.

## Methods

### Participants

Thirteen healthy men (age: 22 ± 2 yr, height: 179 ± 7 cm, body mass: 76 ± 9 kg) volunteered to participate in the present study. Using sample size calculations provided by G*Power, the sample size was calculated a priori to show an effect size of around 1.2 [Cohen’s *d* (Cohen 1988)] for the effect of downhill running on the knee extensors MVIC force (Martin et al. 2004), with an α at 0.05 and a *β* at 0.2. Prior to their inclusion in this study, the participants were screened for the following exclusion criteria: smoking, current medication or drug consumption, and presence of apparent cardiovascular, metabolic, neurologic, or musculoskeletal disease. Furthermore, participants were excluded if they practiced regular physical activity involving substantial eccentric contractions within a six-month period prior to the start date of this study. Recreational running was tolerated except for prolonged (> 10 min) and repeated (more than 2 times per week) downhill running practice. Participants were also excluded if they were already familiarized with other types of eccentric exercises (e.g., strength training) prior to this study. They were also instructed to avoid (i) aspirin, ibuprofen, or other anti-inflammatory drugs; (ii) the consumption of caffeine and/or alcohol on the day of the experiment; and (iii) any strenuous exercise during the 48 h prior to testing. All procedures were approved by the local ethics committee (RCB number ID-RCB: 2019-A03012-55) and this study conformed to the standards set by the Declaration of Helsinki, except for registration in a database.

### Experimental procedures

A pre-post cross-sectional design was used. Each participant attended the laboratory on two separate occasions. Room temperature and humidity were similar across sessions (21 °C, 45% RH). They first came for a familiarization session, and again one week later for the experimental session. During the familiarization session, the participants performed maximal, as well as impulsive, knee extensors voluntary isometric contractions and a single 5-min downhill run, replicating the experimental settings on the same treadmill used for the experimental session. During the experimental session, the participants performed a warm-up consisting of 10 min of light pedaling on a cycle ergometer (Monark 818E, Stockholm, Sweden) and 20 × 1-to-2-s knee extensors voluntary isometric contractions (separated by 10 s each) starting from a self-selected force and progressively increasing until the maximal volitional force was exerted (Coratella et al. 2020). After 5 min, MVIC and RFD were tested before performing a 30-min downhill run at a running speed of 10 km h^−1^ (2.78 m s^−1^) and a slope of − 20% (11.3°) on a motorized treadmill (Medic 2855, Genin Medical, La Roque-d'Anthéron, France). The protocol was chosen to induce significant neuromuscular fatigue, as shown by Martin et al. (2004) and (Khassetarash et al. 2021). Treadmill downhill running was selected since it was illustrated that overground graded running is well replicated on a treadmill (Firminger et al. 2018). After 90 s from the cessation of the downhill running bout, the participants performed the post-test evaluation of MVIC and RFD. This time window of 90 s was kept consistent for all participants, and it was chosen after pilot tests as it allowed enough time to move and install the participant on the isometric chair after the downhill running bout.

### Neuromuscular evaluation

The testing protocol for MVIC and RFD was identical before (PRE) and after (POST). The downhill running session consisted in a 5-s MVIC followed by a series of eight impulsive contractions. Contractions were separated by 5 s of rest. At PRE, two MVICs were performed after having completed the warm-up protocol, and the peak value was retained for further analysis. If the difference between the two MVICs was > 5%, further trials were performed until the difference between two consecutive trials was < 5%.

The instruction given for all MVICs was to contract “as hard as possible,” while for impulsive contractions the participants were asked to extend the knee “as fast as possible” without any countermovement (Varesco et al. 2019). In case of a countermovement (determined by a force drop of 2 N below the baseline right before the impulsive contraction) or pre-tension (determined by a force level ≥ 2 N above the baseline right before the impulsive contraction) the contraction was repeated. The impulsive contractions were also repeated if the force level was < 70% of the MVIC that preceded the series of impulsive contractions (Varesco et al. 2019). All testing protocols were performed with real-time visual feedback.

### Experimental setting

The participants were seated upright with hip and knee angles set at 90° of flexion on an isometric chair, equipped with a calibrated force transducer (Legcontrol, Mtraining, Ecole Valentin, France). The lever arm of the dynamometer was adjusted to firmly attach the leg 3 cm above the medial malleoli with two non-compliant belts. A belt strapped over the waist was used to minimize extraneous movements of the upper body. Passive resting force was subtracted from the signal so that the baseline was set at 0 N.

The EMG activity was recorded from the *vastus lateralis* muscle of the right leg as a surrogate for knee extensors activity (Place et al. 2010; Coratella et al. 2018). Self-adhesive Ag/AgCl surface electrodes (recording diameter = 10 mm, Kendall MediTrace foam electrode, MA) were placed in bipolar configuration over the muscle belly with an interelectrode (center to center) distance of 30 mm (Hermens et al. 2000). A reference (ground) electrode was placed on the right patella. Prior to electrode placement, the skin was shaved, abraded, and gently cleaned with an alcohol swab to lower the impedance. The EMG and force signals were collected simultaneously without analogical filters at a frequency of 2 kHz by PowerLab System (16/30-ML880/P, AD Instruments, Bellavista, Australia) and transferred on a personal computer using Labchart 8 software (ADInstruments) interface. The EMG signals were amplified (gain = 500) using an octal bio-amplifier (ML138, AD Instruments) with a bandwidth frequency range of 10–500 Hz obtained through a digital band-pass filter. EMG data were rectified and smoothed using a moving root mean square (RMS) with a time constant interval of 50 ms (Aagaard et al. 2002; Varesco et al. 2019).

### Additional experiment

To confirm that changes in MVIC and RFD at POST results from the downhill running intervention, eight additional participants were tested as control group (age: 24 ± 5 yr, height: 176 ± 9 cm, body mass: 69 ± 8 kg). The setup and procedures were the same as the testing of the main study. However, these participants performed the neuromuscular evaluations PRE and POST with a resting period of equivalent length to the downhill running intervention.

### Data analysis

**Fig. 1:**
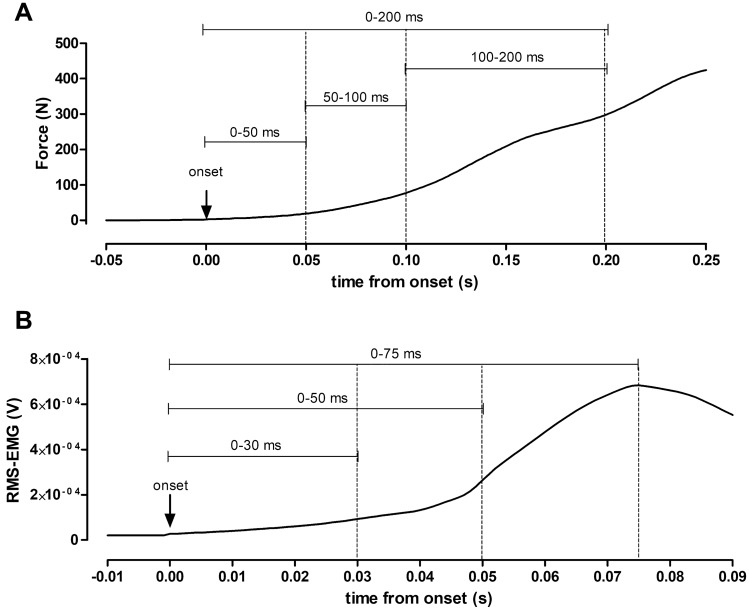
Typical trace of the rate of force development (Panel **A**) and rate of EMG rise (Panel **B**) of a representative participant. Panel **A**: Rate of force development at different time intervals from the onset: 0–50 ms, 50–100 ms, 100–200 ms, and 0–200 ms. Panel **B**: Rate of EMG rise at different time intervals from the onset: 0–30 ms, 0–50 ms, 0–75 ms

For each maximal contraction, MVIC force and the associated RMS EMG signal (EMG_max_) were calculated over the highest 1-s window. For each impulsive contraction, the peak RFD was determined as the maximum value on the force–time derivative curve over a 10-ms period to identify the best five impulsive contractions over all those performed (Varesco et al. 2019). The five contractions retained were averaged for further analysis, while the others were discarded. The onset of the force development was automatically defined as the point at which force exceeded the average resting baseline by ~ 2 N. The onset was also checked visually by an experienced investigator blinded to the condition (PRE or POST). Force was then measured at 25, 50, 100, and 200 ms after the onset (Fig. 1A). RFD was calculated at three time intervals of 0–50 ms (RFD_0–50_), 50–100 ms (RFD_50–100_), and 100–200 ms (RFD_100–200_) from the mean linear slope of the force–time curve (Aagaard et al. 2002; Coratella et al. 2020). The RFD (RFD_0–200_) was also calculated from contraction onset to 200 ms as an overall index of rapid force production ability at PRE and POST. The RER was calculated as the mean linear slope of the RMS EMG–time curve and analyzed at time intervals of 0–30 ms (RER_0–30_), 0–50 ms (RER_0–50_), and 0–75 ms (RER_0–75_) (Fig. 1B). The onset was visually identified on the RMS EMG–time curve as a rise of the RMS EMG signal over the baseline (Varesco et al. 2019). As the RMS EMG signal was nearly silent before the contraction, it was not necessary to set a threshold for noise (Fig. 1B). The long interval for the RER was measured on the first 75 ms of contraction as suggested by (Aagaard et al. 2002). This is also because, for our dataset, a decrease in RMS EMG signal amplitude occurred after ~ 80–100 ms from the onset (Fig. 1B). All RFD parameters are presented in both absolute and normalized to MVIC values. All RER intervals are presented in both absolute and normalized to EMG_max_ values. All data were analyzed offline using Labchart 8 Software (ADInstruments).

### Statistical analysis

Data are reported as mean ± standard deviation. Assumption of normality (Shapiro–Wilk test) was tested for all variables. Generalized estimating equations were employed to account for the unbalanced participant numbers on data relative to baseline (*n* = 13 for the downhill running intervention and *n* = 8 for the control group) (Liang and Zeger 1986). If significant time (PRE vs. POST) × group (downhill running vs. resting) interactions were observed on MVIC and RFD_0–200_, the etiology was investigated using paired samples *t*-tests for normally distributed variables (MVIC, EMG_max_, absolute and normalized RFD windows, normalized RER variables) to compare differences between PRE and POST. For non-normally distributed variables (absolute RER variables), Wilcoxon Signed-Rank Tests were used to compare differences between PRE and POST. As a measure of effect size for parametric tests, Cohen’s *d* was calculated with 95% confidence intervals (CI). Values of ± 0.20, ± 0.50, and over ± 0.80 were considered as small, medium, and large effect, respectively (Cohen 1988). As a measure of the effect size for non-parametric tests, eta squared (*η*^2^) was calculated with 95% CI. Values of ± 0.01, ± 0.06, and over ± 0.14 were considered as small, medium, and large effect, respectively (Cohen 1988). The criterion *α*-level was set to 0.05. The statistical analysis was conducted using IBM™ SPSS™ Statistics (Version 28.0, IBM Corp., Somers, New York, NY).

## Results

Significant time × group interactions were observed for MVIC (χ^2^ (1) = 42.817, *P* < 0.001), normalized peak RFD (χ^2^ (1) = 24.909, *P* < 0.001), RFD_0–200_ (χ^2^ (1) = 5.847, *P* = 0.016), and normalized RFD_0–200_ (χ^2^ (1) = 5.700, *P* = 0.017). For all these parameters, the two groups were similar at PRE (all *P* ≥ 0.375). At POST, the two groups differed with the group of participants who performed the downhill running intervention that presented lower values than the group of participants who rested for equivalent length to the downhill running intervention (all *P* ≤ 0.018) (Table 1). Therefore, only changes in MVIC and RFD at POST for the downhill running group are presented.

**Table 1:** Comparison in neuromuscular changes for the main outcomes in the experimental and control groups

	PRE		POST		Time × group interaction
	CTRL	EXP	CTRL	EXP	
MVIC (N)	550 ± 145	609 ± 172	546 ± 125	458 ± 129^*,$^	< 0.001
peak RFD (N s^−1^)	7978 ± 2674	8267 ± 3274	7817 ± 2404	7276 ± 2893	0.981
RFD_0–200_ (N s^−1^)	2739 ± 794	2463 ± 714	2730 ± 837	2070 ± 795^*,$^	0.016
peak RFD/MVIC [N･(N s)^−1^]	14.7 ± 3.7	13.8 ± 3.2	15.1 ± 4.5	17.0 ± 5.2^*,$^	< 0.001
RFD_0–200_/MVIC [N･(N s)^−1^]	5.0 ± 1.1	4.0 ± 0.4	5.2 ± 1.6	4.6 ± 0.7^*,$^	0.017

*EXP* experimental group performing 30 min of downhill running (speed: 10 km h^−1^; slope: − 20%), *CTRL* control group that rested for an equivalent length to the downhill running intervention, *MVIC* maximal voluntary isometric contraction force, *peak RFD* peak rate of force development (peak RFD), *RFD*_*0–200*_ rate of force development for the time window 0–200 ms

*Time differences between PRE and POST by means of generalized estimated equations (*P* < 0.05)

^$^Group differences at the same time point by means of generalized estimated equations (*P* < 0.05). Statistical analysis was performed on data expressed relatively to baseline

**Fig. 2:**
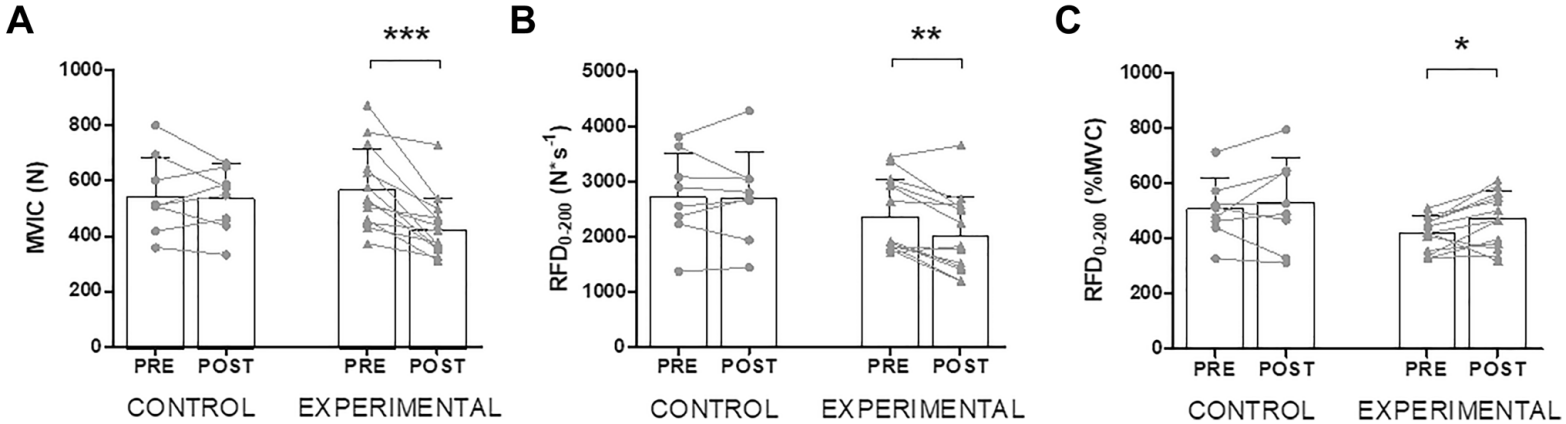
Changes before (PRE) and after (POST) 30 min of rest (CONTROL) or a 30-min downhill running bout (EXPERIMENTAL; speed: 10 km h^−1^; slope: − 20%) in the maximal voluntary isometric contraction force (MVIC; Panel **A**), absolute (Panel **B**), and relative (Panel **C**) rate of force development for the time window 0–200 ms (RFD_0–200_). Values are means ± standard deviations. Asterisks denote time differences between PRE and POST by means of paired samples *t*-tests: *P < 0.05; **P < 0.01; ***P < 0.001

For the group of participants who performed the downhill running intervention, at POST, MVIC and RFD_0–200_ decreased by ~ 25% [*d* = − 1.09 (95% CI − 1.88/− 0.24); *P* < 0.001; Fig. 2A] and ~ 15% [*d* = − 0.50 (− 1.26/0.30); *P* = 0.001; Fig. 2B], respectively. When normalized to the corresponding MVIC, RFD_0–200_ was ~ 11% greater at POST compared to PRE [*d* = 0.71 (− 0.10/1.48); *P* = 0.024; Fig. 2C]. Finally, no significant differences were found from PRE to POST for peak RFD (PRE = 7866 ± 3283 N s^−1^; POST = 7284 ± 2655 N s^−1^; *d* = 0.43 (− 0.15/0.10); *P* = 0.072) and for time to peak RFD (PRE = 0.054 ± 0.010 s; POST = 0.060 ± 0.015 s; *d* = − 0.39 (− 0.95/0.18); *P* = 0.965). For the group of participants who rested for equivalent length to the downhill running intervention, no changes were observed for MVIC, RFD_0–200_, and RFD_0–200_ normalized to MVIC (all *P* > 0.05; Fig. 2).

For the group of participants who performed the downhill running intervention, EMG_max_ showed a significant decrease of ~ 22% at POST [PRE: 0.28 ± 0.18 mV; POST: 0.22 ± 0.14 mV; *d* = − 0.37 (− 1.13/0.42); *P* = 0.038]. Absolute RER of the *vastus lateralis* muscle did not change at any time interval: 0–30 ms [PRE: 4.93 ± 2.84 mV; POST: 5.35 ± 2.64 mV; *η*^*2*^ = 0.086 (− 0.69/0.85); *P* = 0.311], 0–50 ms [PRE: 5.47 ± 3.64 mV; POST: 5.34 ± 2.84 mV; *η*^*2*^ = 0.017 (− 0.75/0.79); *P* = 0.650], or 0–75 ms [PRE: 4.03 ± 3.12 mV; POST: 3.89 ± 1.90 mV; *η*^*2*^ = 0.023 (− 0.55/0.99); *P* = 0.600]. Similar results were observed when RER was expressed as %EMG_max_: RER_0–30_ [PRE: 2203 ± 1138%; POST: 3311 ± 2618%; *d* = 0.55 (− 0.25/1.31); *P* = 0.090], RER_0–50_ [PRE: 2278 ± 1147%; POST: 3052 ± 2037%; *d* = 0.47 (− 0.32/1.23); *P* = 0.153], and RER_0–75_ [PRE: 1504 ± 791%; POST: 2213 ± 1344%; *d* = 0.64 (− 0.17/1.41); *P* = 0.079].

**Fig. 3:**
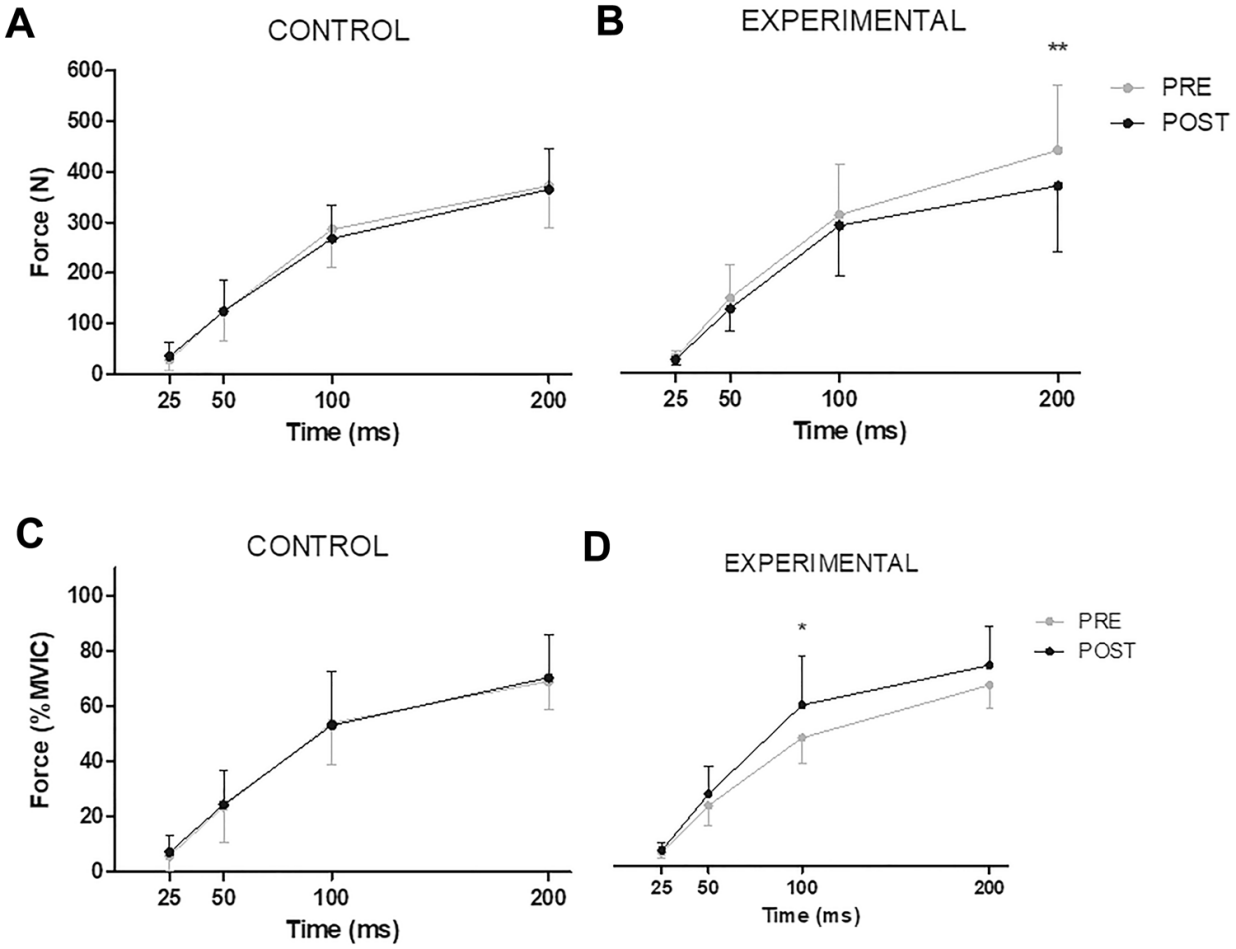
Changes in the absolute force values at different time points (Top panels) and normalized to maximal voluntary isometric contraction force values (MVIC; Bottom panels) before (PRE) and after (POST) 30 min of rest (CONTROL, Panels **A** and **C**) or a 30-min downhill running bout (EXPERIMENTAL; speed: 10 km h^−1^; slope: − 20%, Panels **B** and **D**). Values are means ± standard deviations. Asterisks denote time differences between PRE and POST by means of paired samples *t*-tests: *P < 0.05; **P < 0.01

Figure 3A and B shows the force values at different time points from the onset of the contraction, respectively. Only for the group of participants who performed the downhill running intervention, force at 200 ms decreased by ~ 16% at POST [*d* = − 0.54 (− 1.30/0.26); *P* = 0.008], while no differences were observed at 25 ms [*d* = − 0.42 (− 1.18/0.37); *P* = 0.227], 50 ms [*d* = − 0.38 (− 1.14/0.41); *P* = 0.203] or 100 ms [*d* = − 0.21 (− 0.97/0.57); *P* = 0.254]. When normalized to MVIC (Fig. 3C and D), the force at 100 ms increased by ~ 20% at POST [*d* = 0.83 (0.005/1.60), *P* = 0.016]. No differences were observed for normalized data at 25 ms [*d* = 0.37 (− 0.42/1.13); *P* = 0.267], 50 ms [*d* = 0.49 (− 0.30/1.25); *P* = 0.117] or 200 ms [*d* = 0.61 (− 0.19/1.38); *P* = 0.088]. No changes were observed for the group of participants who rested for equivalent length to the downhill running intervention (all *P* > 0.05).

**Fig. 4:**
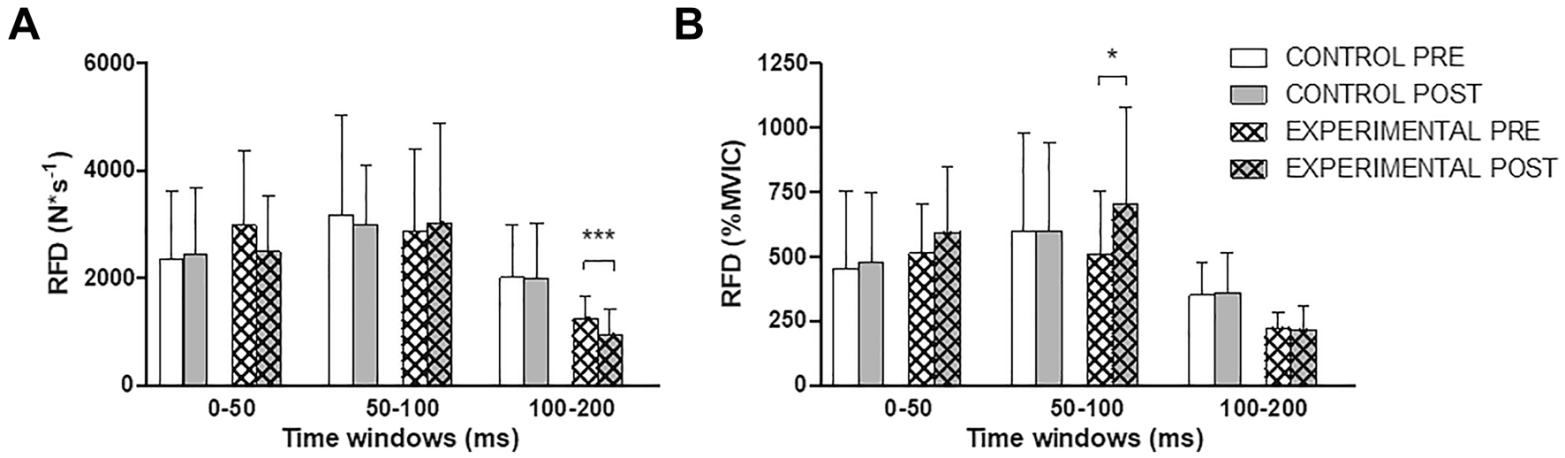
Changes in the rate of force development (RFD) at different time windows expressed as absolute values (Panel **A**) and normalized to maximal voluntary isometric contraction force (MVIC) values (Panel **B**) before (PRE) and after (POST) 30 min of rest (CONTROL) or a 30-min downhill running bout (EXPERIMENTAL; speed: 10 km h^−1^; slope: − 20%). Values are means ± standard deviations. Asterisks denote time differences between PRE and POST by means of paired samples *t*-tests: *P < 0.05; ***P < 0.001

Figure 4A shows the RFD at different time windows from the onset of the contraction. No changes were observed for the group of participants who rested for equivalent length to the downhill running intervention (all *P* > 0.05). For the group of participants who performed the downhill running intervention, RFD_100–200_ was ~ 25% lower at POST compared to PRE [*d* = − 0.70 (− 1.47/0.11); *P* < 0.001]. No differences were observed for RFD_0–50_ [*d* = − 0.40 (− 1.16/0.39); *P* = 0.125] or RFD_50–100_ [*d* = 0.09 (− 0.68/0.86), *P* = 0.599]. When normalized to MVIC (Fig. 4B), no differences were observed for the group of participants who rested for equivalent length to the downhill running intervention (all *P* > 0.05). Conversely, for the group of participants who performed the downhill running intervention, only RFD_50–100_ increased by ~ 26% from PRE to POST [*d* = 0.61 (− 0.19/1.38); *P* = 0.030]. No differences were observed for RFD_0-50_ [*d* = 0.35 (− 1.12/0.43); *P* = 0.179] or RFD_100–200_ [*d* = 0.07 (− 0.84/0.70); *P* = 0.763].

## Discussion

The current study examined for the first time the capacity of the knee extensors to rapidly produce force after a 30-min eccentric-biased downhill run. The downhill running bout induced a drop in MVIC and impaired the overall ability to rapidly develop force, as indicated by the reduction in RFD_0–200_. When analyzing the different RFD time windows, we observed a reduction in the 100–200 ms phase, while the early phase of the RFD remains unchanged. This latter was accompanied by a lack of change in the absolute RER. Together, these data suggest that a fatiguing downhill run affects the force-generating capacity and the RFD during isometric impulsive contractions through structural rather than neural impairments.

### Maximal isometric force

After the 30-min downhill run, MVIC decreased by ~ 25%, which was consistent with the values reported in the literature (Martin et al. 2004; Giandolini et al. 2016a; Ehrström et al. 2018; Lemire et al. 2020; Khassetarash et al. 2021). The force loss after downhill running has been related to impairments in both neural and contractile components (Giandolini et al. 2016a; Ehrström et al. 2018; Khassetarash et al. 2021). Specifically, the former seems attributable to a deficit in muscle voluntary activation caused by several factors, such as neurobiological alterations in the brain and/or changes in the intrinsic properties of the motoneuron pool (Gandevia 2001). In contrast, the latter appear primarily due to impairments in one or more steps involved in the excitation–contraction coupling, such as decoupling at the T-tubule–sarcoplasmic reticulum interface and/or a decreased Ca^2+^ release from the sarcoplasmic reticulum (Giandolini et al. 2016b; Bontemps et al. 2020). Furthermore, EMG_max_ was lower at POST compared to PRE, and this has been associated with a reduced firing rate of high threshold motor units (Balshaw et al. 2017).

### Maximal rate of force development

RFD_0–200_ decreased by ~ 15% after the 30-min downhill run, which is less than the ~ 25% drop observed in MVIC. Consequently, normalized RFD (%MVIC) was greater, indicating that downhill running-induced fatigue affected the maximal force capacity to a greater extent compared to RFD. Previously, Maeo et al. (2017) evaluated the knee extensors MVIC and RFD responses following 45 min of downhill running at ~ 10 km h^−1^ and − 15% slope. Twenty-four hours after the exercise, the authors observed a decrease of ~ 16% and ~ 20% in MVIC and RFD_0–200_, respectively. However, neither the different phases of the force–time curve of RFD nor the RER were investigated. Furthermore, RFD was calculated on the rising in force during MVIC and attempting to measure RFD during an MVIC may have resulted in an underestimation of RFD (Maffiuletti et al. 2016). These limitations impede direct comparisons with the current study. Recently, using a similar protocol, Khassetarash et al. (2021) found a 16% decrease in MVIC, while we found much larger impairments (− 25%). Those authors did not evaluated RFD; however, they studied the possible etiology for the MVIC force loss using the interpolated twitch technique with electrical stimulations. They found impairments in both voluntary activation (~ 8.6%) and artificially evoked force responses (~ 4.9%). Therefore, it is possible that, in our study, the downhill running bout induced impairments in both maximal voluntary activation and contractile function. During a 5-s MVIC, the time is sufficient to recruit most of the motor unit pool (De Luca 1985; Maffiuletti et al. 2016). It is then possible that impairment in maximal voluntary activation or contractile function would manifest to a larger extent during MVIC than during RFD. While during RFD contraction it would be possible to detect the rapid activation of the muscle rather than the maximal one (Maffiuletti et al. 2016; D’Emanuele et al. 2021).

### Early and late phases of the rate of force development

No changes were observed for both the early phase of the RFD and the absolute RER, suggesting no detectable alterations in the generation and/or transmission of firing bursts along the neuromuscular pathway (Klass et al. 2008; Del Vecchio et al. 2019).

In the present study, the downhill running-induced fatigue altered the late phase of the RFD, as indicated by a decrease in RFD_100–200_. Several possible mechanisms may have contributed to the decrease in the late phase of the RFD. First, the responsiveness of a muscle to high firing rates from the motoneuron pool depends on both the Ca^2+^ sensitivity and the number of actin-myosin bridges available for contractions (Allen et al. 2008). The repetitive eccentric muscle actions that occur during the braking phase of each landing step of a downhill run (Vernillo et al. 2017) were shown to cause reductions in the Ca^2+^ sensitivity, actin–myosin coupling (Giandolini et al. 2016b; Bontemps et al. 2020), and contiguity of the Z-lines of the sarcomere (Féasson et al. 2002)﻿. These structural alterations within the muscle likely affected the interface sarcoplasmic reticulum/T-tubule (Giandolini et al. 2016b), negatively influencing the late phase of the RFD after downhill running. Second, the decrease in the late phase of the RFD could have also been modulated by an alteration in tendon stiffness. Indeed, repetitive eccentric muscle actions were shown to induce considerable stress on the passive structures of the muscle (i.e., muscle connective tissue and tendons) (Reeves and Narici 2003). During a downhill run this could have led to repetitive elongations of the knee extensors muscle–tendon unit, reducing stiffness and consequently increasing compliance (Guilhem et al. 2011). The reduced stiffness of the muscle–tendon unit could then explain the observed decreased late-phase RFD after the downhill running with an altered force transmission (Hannah and Folland 2015). Third, downhill running may have induced changes in the muscle architecture. Fatiguing tasks may result in a decreased fascicle length (Csapo et al. 2011), and considering that fascicle length is a possible predictor of the RFD late phase (Coratella et al. 2020), it would then be possible that downhill running may have affected the fascicle length. However, it is acknowledged that this remains to be proven in future investigations. Our results suggest that the ability to rapidly activate the muscle after downhill running was preserved, as shown by the absence of significant change in RER and RFD_0–50_ from PRE to POST. Although speculative, it is possible that downhill running would only cause structural alterations (or a reduction in the total number of muscle fibers activated during the rapid contractions), thereby impairing RFD ability.

## Limitations

Some possible limitations could have influenced the outcomes assessed in the present study. First, our POST measurements were collected after 90 s of recovery. As recovery kinetics at the level of the central nervous system is rapid (Kennedy et al. 2016; Mira et al. 2017; Vernillo et al. 2018; Krüger et al. 2019; Koral et al. 2020; Vernillo et al. 2020b; Ducrocq et al. 2021), we cannot rule out that the capacity to deliver maximal discharge frequency and incidence of doublet discharge during fast contractions (i.e., the neural factors influencing RFD) has been underestimated in the present study. Second, RFD contractions also depend on the ability of the individual to perform impulsive contractions. We limited its possible effect by performing a familiarization session where participants were instructed by an expert investigator to perform impulsive contractions and strongly encouraged during the experiments. Finally, trains of high frequency evoked contractions would have provided additional information independent of any volitional input (to better discern voluntary vs. involuntary muscle rapid activation capacity). However, these procedures are painful and could increase the risk of injury (Millet et al. 2012).

## Conclusions

The present findings showed that RFD is altered after a single bout of downhill running. Specifically, we observed that only the late phase of the force–time curve during impulsive contractions was altered. It seems that a bout of downhill running influenced more the structural than the neuromuscular factors, as indicated by the drop in the late but not early phase of RFD. Since downhill running is an essential component of trail running performance, these findings may help explain evidence of neuromuscular alterations in trail runners and following prolonged duration races wherein cumulative eccentric loading is high.

## Data Availability

Derived data supporting the findings of this study are available on request from the corresponding author.

## Abbreviations

CI: 95% Confidence intervals
EMG: Electromyography
MVIC: Maximum voluntary isometric contraction
POST: Neuromuscular evaluation performed after the fatiguing exercise
PRE: Neuromuscular evaluation performed before the fatiguing exercise
RER: Rate of electromyography signal rise
RFD: Rate of force development
RFD_0–50_: Rate of force development calculated at the time interval of 0–50 ms
RFD_50–100_: Rate of force development calculated at the time interval of 50–100 ms
RFD_100–200_: Rate of force development calculated at the time interval of 100–200 ms
RMS: Root mean square
